# Carbon Materials as a Conductive Skeleton for Supercapacitor Electrode Applications: A Review

**DOI:** 10.3390/nano13061049

**Published:** 2023-03-14

**Authors:** Yedluri Anil Kumar, Ganesh Koyyada, Tholkappiyan Ramachandran, Jae Hong Kim, Sajid Sajid, Md Moniruzzaman, Salem Alzahmi, Ihab M. Obaidat

**Affiliations:** 1 Department of Chemical & Petroleum Engineering, United Arab Emirates University, Al Ain 15551, United Arab Emirates; 2 National Water and Energy Center, United Arab Emirates University, Al Ain 15551, United Arab Emirates; 3 Department of Chemical Engineering, Yeungnam University, 214-1 Daehak-ro 280, Gyeongsan 712-749, Gyeongbuk-do, Republic of Korea; 4 Department of Physics, College of Science, United Arab Emirates University, Al Ain 15551, United Arab Emirates; 5 Department of Chemical and Biological Engineering, Gachon University, 1342 Seongnam-daero, Seongnam-si 13120, Gyeonggi-do, Republic of Korea

**Keywords:** carbon materials, supercapacitors, nanoarchitectures, energy storage

## Abstract

Supercapacitors have become a popular form of energy-storage device in the current energy and environmental landscape, and their performance is heavily reliant on the electrode materials used. Carbon-based electrodes are highly desirable due to their low cost and their abundance in various forms, as well as their ability to easily alter conductivity and surface area. Many studies have been conducted to enhance the performance of carbon-based supercapacitors by utilizing various carbon compounds, including pure carbon nanotubes and multistage carbon nanostructures as electrodes. These studies have examined the characteristics and potential applications of numerous pure carbon nanostructures and scrutinized the use of a wide variety of carbon nanomaterials, such as AC, CNTs, GR, CNCs, and others, to improve capacitance. Ultimately, this study provides a roadmap for producing high-quality supercapacitors using carbon-based electrodes.

## 1. Introduction

The development of diverse energies, including solar energy, wind energy, tidal energy, and nuclear energy, is driven by the conflict between human dependence on energy and the burning of fossil fuels [1,2]. However, the sporadic energy supply from these systems necessitates an effective storage mechanism for safe and constant power acquisition. Supercapacitors and lithium-ion batteries (LIB) have been mentioned as prospective electrical-energy-storage technologies up to this point [3]. All of these have a strong connection to the advancement of improved energy-storage technology. The most promising energy-storage technologies to date, for electrochemical-energy-storage systems, are various types of rechargeable batteries and supercapacitors [4,5]. Biomass is one of the most abundant renewable resources on the planet. Biomass has been studied and exploited for several applications, including the collection of carbon dioxide (CO_2_) [6], the storage of hydrogen [7], dye-sensitized solar cells [8], water treatment [9], and energy storage [10].

Alternative power technologies, for example electrochemical-energy systems, such as batteries, fuel cells, and rechargeable batteries, have grown more popular due to environmental pollution and the depletion of fossil fuels. These are small, portable gadgets with the ability to transform and store energy from other sources [11]. Supercapacitors, which polarize the electrolyte to store energy, are promising electrochemical devices. They offer higher energy densities and better rate performance over time (around 1000 cycles) than rechargeable batteries. They have attracted considerable interest because of their excellent safety, long lifespan, short charging time, and high power density. Supercapacitors can charge and discharge very quickly because of their electrode materials [12,13,14]. Producing supercapacitor devices with high power densities without sacrificing their energy density, long lifespan, quick charge/discharge, and eco-friendly qualities, however, is still a difficult task. Moreover, because of their poor energy density, difficult manufacturing process, and high cost, supercapacitors have encountered significant challenges in finding practical uses [15,16,17]. Supercapacitors can be divided into two types, based on how they store their charge, which also influences how densely they can store power [18]. Pseudocapacitance and electrical double-layer (EDLC) capacitance differ greatly from one another. Supercapacitors (SCs) have come a long way in recent years thanks to significant advancements in research and technology as well as increased energy density values, making them a viable alternative to traditional batteries [19,20,21,22]. The performance of SCs is significantly improved by the electrode material used, which is the corresponding key for SCs [23]. The choice of electrode material often depends on the various charge-storage techniques [24].

Carbon has been the most extensively employed electrode material recently across all supercapacitor electrode materials. Carbon black, aerogel particles, activated carbon, and carbon cloth have all been used to create double-layer capacitor electrodes [25,26,27,28]. Carbons are among the most interesting electrically conductive materials because of their low energy density, high stability, and satisfactory corrosion resistance [14,15]. Additionally, by employing oxidizing chemicals during heated processes, a process known as activation, it is possible to easily tailor the porosity and shape of carbons. For example, very porous carbon material with a vast surface area of up to 2000 m^2^ g^−1^ has been produced [29]. Supercapacitors composed of carbon materials have numerous distinct benefits over other forms of energy storage, such as fuel cells and lithium batteries [30,31]. Carbon nanotubes (CNTs) are a class of carbon materials that exhibit excellent electron transport, high electrical conductivity, and accessibility to electrolytes. CNTs are therefore regarded as a potential material for the production of electrodes. Another attractive electrode material is graphene, which has remarkable conductivity and a large specific surface area [32,33]. Likewise, carbon nanoflowers, carbon quantum dots, graphene sheets, etc., also exhibit good characteristics. The composite of carbon structures results in high surface area, conductivity, and charge-storing capacities [34]. In recent years, the study of graphene, a two-dimensional carbon substance with exceptional electrical and mechanical characteristics, has accelerated significantly. Due to their superior mechanical qualities, graphene and carbon nanotubes are frequently employed as electrode materials for flexible electronics [35,36,37]. Micropores are an ideal component for studying how pore size influences electrochemically efficient electrodes [38]. The specific capacitance of carbon materials can be somewhat improved by increasing the specific surface area, altering the pore structure, and improving the wettability of the electrolyte [39].

## 2. Role of Carbon-Based Materials in Energy-Storage Devices

Because of their exceptional and improved characteristics as well as their programmable surface chemistry, carbon-based nanomaterials can be employed to construct effective high-energy and high-power energy-storage devices [40]. Due to the enormous demand for energy and the depletion of fossil fuel resources, researchers have shown a significant degree of interest in developing materials with improved electrochemical capabilities. Carbon-based materials, such as carbon nanotubes (CNTs), graphene (GO and rGO), activated carbon (AC), and conducting polymers (CPs), have attracted a lot of attention due to their remarkable thermal, electrical, and mechanical properties [41]. As essential electrode components—active materials, conductive additives, and buffering frameworks—carbon materials demonstrate their significance in electrochemical energy-storage (EES) systems. It is necessary to rationally design functional carbon materials on the basis of a thorough understanding of structure–property relationships in order to meet the demands of the rapidly expanding markets for EES, particularly the electric- vehicle and large-scale-energy-storage markets. Dimensionality variations and hybridizations of carbon materials are key factors in improving the electrochemical performances of EES devices [42]. Carbon materials can be efficiently combined with a variety of other materials or can be made with various microstructures to create stretchy energy-storage devices [43]. The discovery of carbon-based nanomaterials that have exceptional energy conversion and storage capabilities holds the possibility of opening up new avenues for their continued advancement.

## 3. Graphene in Energy-Storage Devices

**Figure 1:**
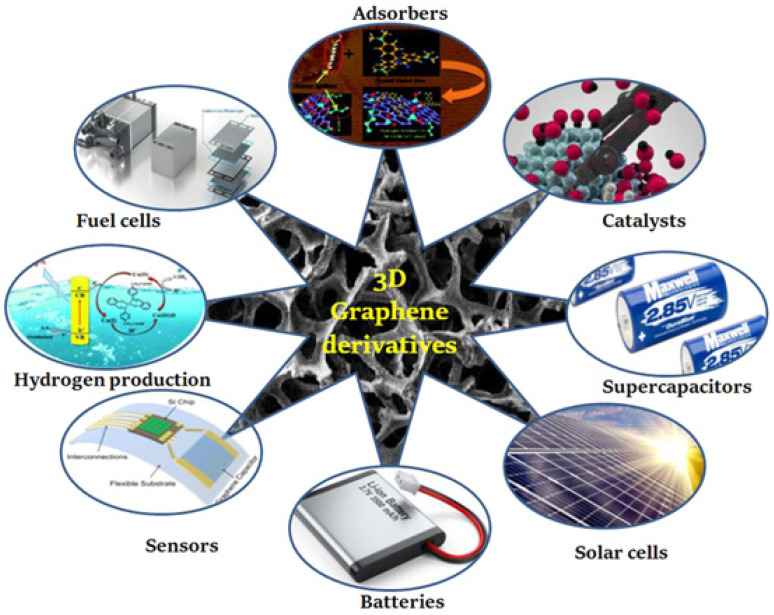
Diagram illustrating the uses of graphene and graphene derivatives in numerous battery storage and conversion technologies. Reproduced with permission from [46].

Energy-storage technologies such as batteries and supercapacitors [6,7] are undergoing extensive study in the search for ways to boost their energy density in order to keep up with the rapidly increasing types of renewable energy sources. Graphene has advantages over other materials, such as its extraordinary mechanical stability, high electrical conductivity, high thermal conductivity, and ability to absorb solar radiation [44,45]. Graphene is also well suited to the development of energy-storage systems. This is possible, especially if the graphene sheets have come into contact with metal oxide, which leads to a limited amount of sheet restacking. Figure 1 shows the uses of graphene and graphene derivatives in numerous battery storage and conversion technologies.

Graphene’s interconnected networks are highly conductive, which adds to the appeal of using them as energy-storage applications. However, graphene is rarely employed as the support material in proton exchange membrane (PEM) fuel cells. Graphene is widely used as the cathodic and anodic material in batteries such as lithium (Li) batteries. Both double-layer capacitors and pseudocapacitors in supercapacitors use graphene as the electrode material [47]. After N2H4 vapor reduction, spray-drying graphene oxide at low temperatures, and annealing at very high temperatures, Chen et al. [48] produced reduced graphene flowers.

## 4. Zero-, One-, Two-, and Three-Dimensional Carbon Materials as an Electrode for Supercapacitors

**Figure 2:**
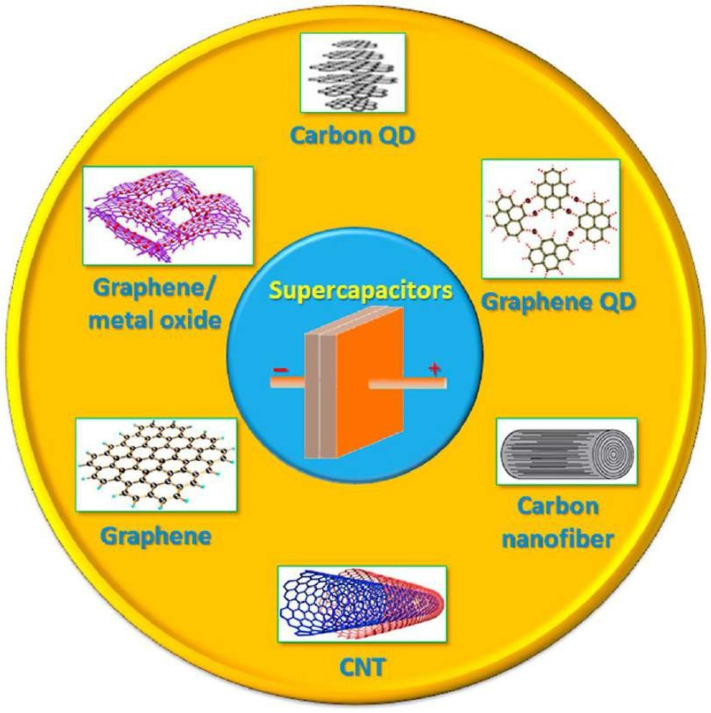
One- to two-dimensional nanostructured carbon-based materials. Reproduced with permission from [49].

Several types of carbon nanomaterials, such as AC, CNTs, GR, CNCs, and many others, are being studied to increase the capacitance. Figure 2 represents the one- to two-dimensional nanostructured carbon-based materials.

Because of their natural richness in a wide range of ways, and their low cost, these carbon nanomaterials are perfect contenders for supercapacitor applications. Furthermore, the surface area and conductivity of carbon can be easily modified. The performance of carbon-based supercapacitors is now being improved through extensive research employing a variety of carbon compounds. In addition, emerging fabrication technologies have allowed the investigation of novel carbon nanomaterials of different dimensions, such as zero-dimensional quantum dots (QDs) and one- and two-dimensional nanostructures.

### 4.1. Zero-Dimensional Materials

Nanomaterials with zero dimensions (0D) have been recognized as the forerunners of nanotechnology [50]. Carbon dots are newly developed carbon nanomaterials with nearly spherical shapes and incredibly small particle sizes, frequently less than 10 nm. They have a conjugated sp^2^/sp^3^ core with a high concentration of functional groups, including carboxyl, hydroxyl, and aldehyde. Carbon QDs have ultra-small sizes in comparison to other carbon materials, granting them uniform dispersion, superior electron transfer/reservoir capabilities, photoluminescent properties, and increased quantum effects [51].

**Figure 3:**
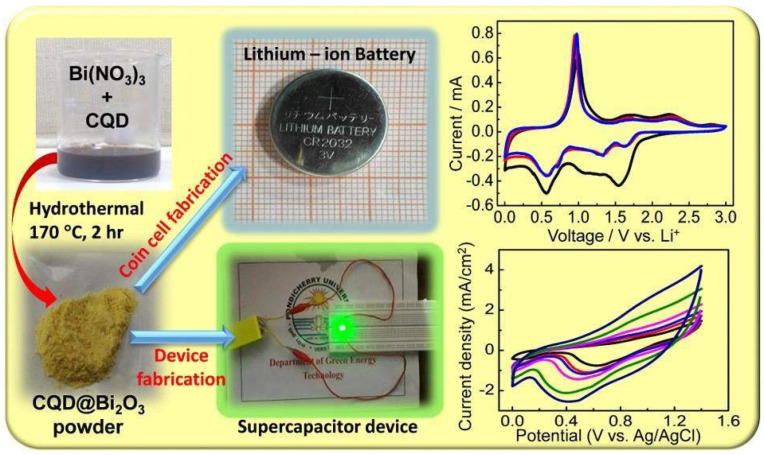
CQD–Bi2O3 composite used as a supercapacitor electrode. Reproduced with permission from [52].

**Figure 4:**
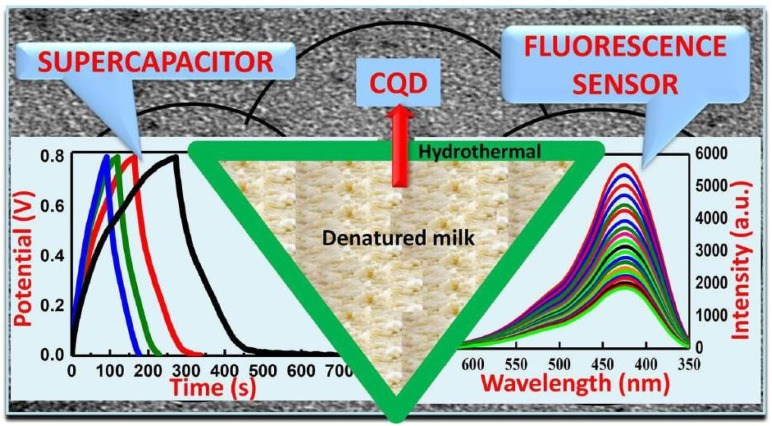
Denatured milk carbon quantum dots for effective chromium-ion detection and supercapacitor applications [52].

The CQD–Bi2O3 composite has performed well as an electrode material in a lithium-ion battery, delivering a discharge capacity of 1500 mA h g^−1^ at a 0.2 C rate. The CQD–Bi2O3 composite electrode’s supercapacitor capabilities demonstrated good reversibility and high potential material for supercapacitors. Figure 3 and Figure 4 show the CQD–Bi2O3 composite used as a supercapacitor electrode and denatured-milk carbon quantum dots used for effective chromium-ion detection, respectively.

### 4.2. One-Dimensional Materials

**Figure 5:**
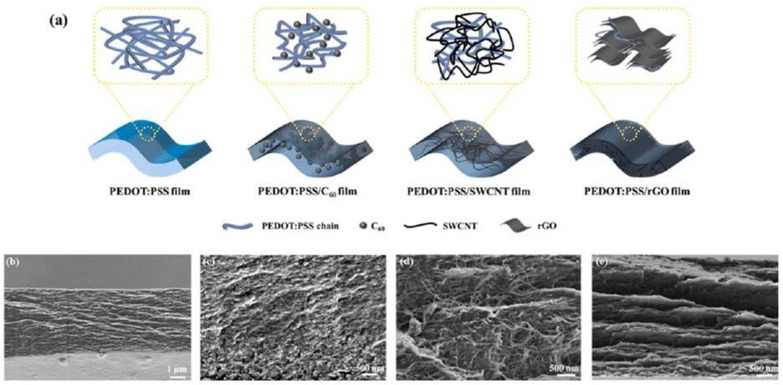
(**a**) The schematic diagram for composites of PEDOT:PSS with nanostructured carbon materials; Cross-sectional SEM images of (**b**) PEDOT:PSS, (**c**) PEDOT:PSS/C_60_, (**d**) PEDOT:PSS/SWCNT, and (**e**) PEDOT:PSS/rGO films. Reproduced with permission from [57].

One-dimensional energy-storage technologies, particularly 1D supercapacitors (SCs), have recently emerged as promising applicants [53,54]. The scientific community has focused heavily on using 1D carbon materials to develop flexible and wearable energy-storage devices [55], such as graphene-based fibers, carbon fibers, and CNTs. One-dimensional carbon compounds have a high aspect ratio and outstanding electron-transport characteristics. They can efficiently speed up the kinetics of electrochemical reactions, provide a large specific surface area, and shorten the transmission/diffusion paths of electrons and ions when used as the electrode materials for SCs. There are four key research directions to follow: improving mechanical properties, producing improved electrochemical performance, enabling the integration of various devices, and displaying multifunctionality [56]. Furthermore, in recent studies, the combination of graphene and CNT fibers in hybrid materials improved mechanical flexibility, electrical and thermal conductivities, and charge-transport properties because of the strong π–π stacking as compared to any hybrid component alone. Cheng et al. [57,58] produced CNTs using a CVD technique over 2D graphene sheets and created CNTs-G hybrid fibers that displayed continuous CV characteristics, even after 200 bending cycles, and an area capacitance of 1.2–1.3 mF cm^−2^. Figure 5 represents a schematic diagram for composites of PEDOT:PSS fabricated with nanostructured carbon materials.

Na2Ti3O7 and single-walled carbon nanotubes were used to develop an electrode material for energy-storage devices by Prabhakar et al. [59]. Due to the produced composite electrode’s mesoporous structure, an energy density of 8.75 Wh kg^−1^ at a power density of 4500 W kg^−1^ was achieved and shown to be significant [60,61,62,63].

### 4.3. Two-Dimensional Materials

**Figure 6:**
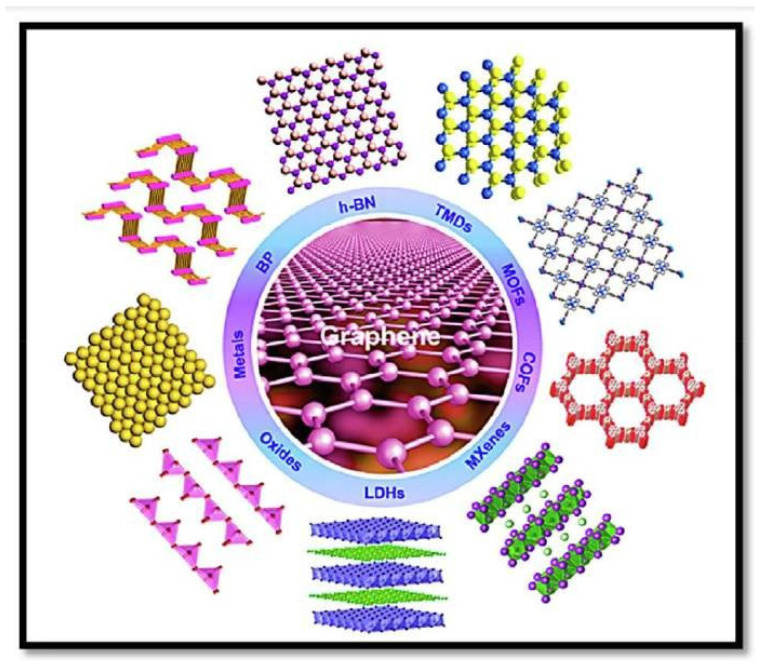
Illustration of various types of two-dimensional nanomaterials for electrode materials in supercapacitors. Reproduced with permission from [64].

Novel two-dimensional (2D) materials have shown various uses in a range of fields due to their exceptional optical, physical, chemical, and electrical properties. Metal–organic frameworks, for example, are employed as electrodes in supercapacitors [64,65] and are depicted in Figure 6.

Ayman et al. [66] produced an electrode from MXene and its composites with cobalt ferrite [CoFe2O4] nanoparticles (CoF NPs). According to electrochemical experiments, the composite (CoF/MXene) can offer greater electrochemical properties than either ferrite or MXene on their own. At 1 A g^−1^, the highest specific capacitance (Csp) of CoF NPs, MXene, and CoF/MXene composites, respectively, was found to be around 594, 1046.25, and 1268.75 F g^−1^.

Recent developments in carbon materials based on metal–organic frameworks (MOFs) have shown promise due to their distinctive characteristics [67,68]. Ding et al. [69] prepared 2D ZIF-8-derived carbon nanosheets (ZCNs). The ZCNs materials resulted in significant ion-accessible surface areas and high ion-/electron-transport rates.

**Figure 7:**
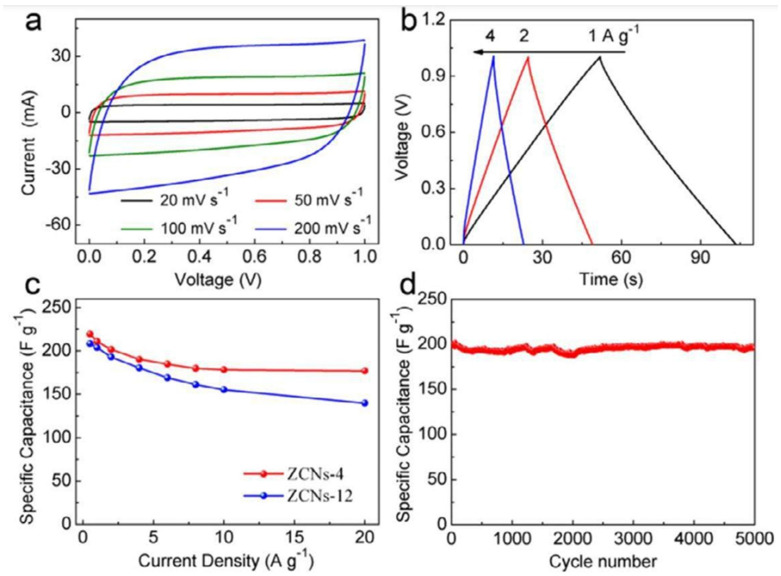
Electrochemical performance of ZCNs electrodes: (**a**) CV curves of ZCNs-4 at various scanning rates; (**b**) galvanostatic charge/discharge curves under various current densities for ZCNs-4; (**c**) specific capacitance dependence on current density of ZCNs-4 and ZCNs-12; (**d**) long-term cycle stability of ZCNs-4 at a current density of 2 A g^−1^. Reproduced with permission from [69].

Figure 7 shows electrochemical characteristics of ZCNs electrodes in 6M KOH. The curve in Figure 7a is rectangular and exhibits good supercapacitive behavior even at 200 mVS^−1^. Figure 7b shows triangularshaped curves without the potential drop, indicating good charge transfer within ZnS-4 electrodes. The computed specific capacitances of the ZCNs-4 and ZCNs-12 electrodes at various current densities are contrasted in Figure 7c. ZCNs-4 shows a high capacitance retention of over 97% after 5000 cycles at 2 A g^−1^ (Figure 7d).

### 4.4. Three-Dimensional Materials

**Figure 8:**
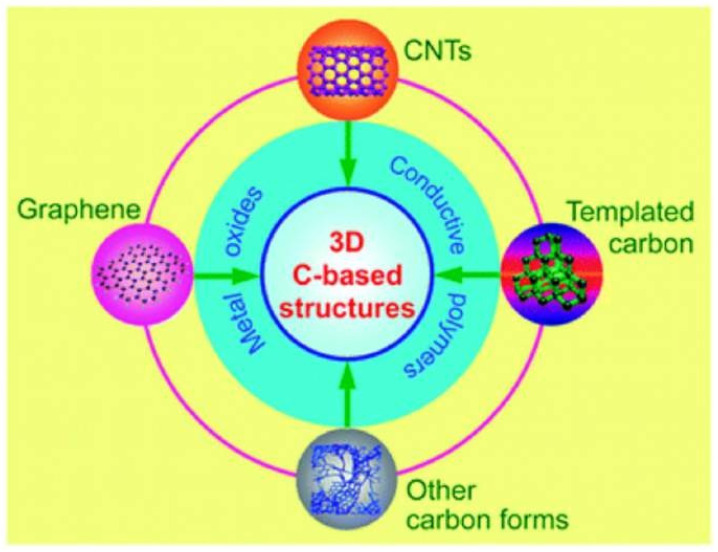
Different kinds of carbon combined with 3D carbon-based nanostructures and pseudo- active materials. Reproduced with permission from [72].

For high-performance supercapacitors, 3D carbon-based nanostructures are now a hot topic [70]. Three-dimensional carbon-based nanostructures generate hierarchical porous channels and have stronger electrical conductivity due to the structural interconnectivity. They also have better structural–mechanical stability. Since each of the qualities of the individual building blocks can be significantly improved if an appropriate nanostructure is chosen, the design and optimization of 3D carbon-based nanostructures are unquestionably crucial [71]. Figure 8 illustrates CNTs-based networks, graphene-based structures, hierarchically porous carbon nanostructures, and even more intricate carbon-based three-dimensional arrangements.

Sun et al. [72] fabricated electrodes with nitrogen-rich 3D carbon material, and this resulted in a strong rate capacitance of 412 F g^−1^. Zhi et al. [73] synthesized electrode material from chicken eggshell membrane, and the prepared 3D carbon fibers resulted in 10 wt% oxygen and 8 wt% nitrogen, a surface area of 221 m^2^/g, and, in a three-electrode system, specific capacitances of 297 F g^−1^ for basic electrolytes and 284 F g^−1^ for acidic electrolytes. Lin et al. [74] synthesized hierarchical porous carbon based on lignin (LHPC). The final LHPC was a 3D network decorated with carbon wall-mounted hierarchical mesopores and micropores.

**Figure 9:**
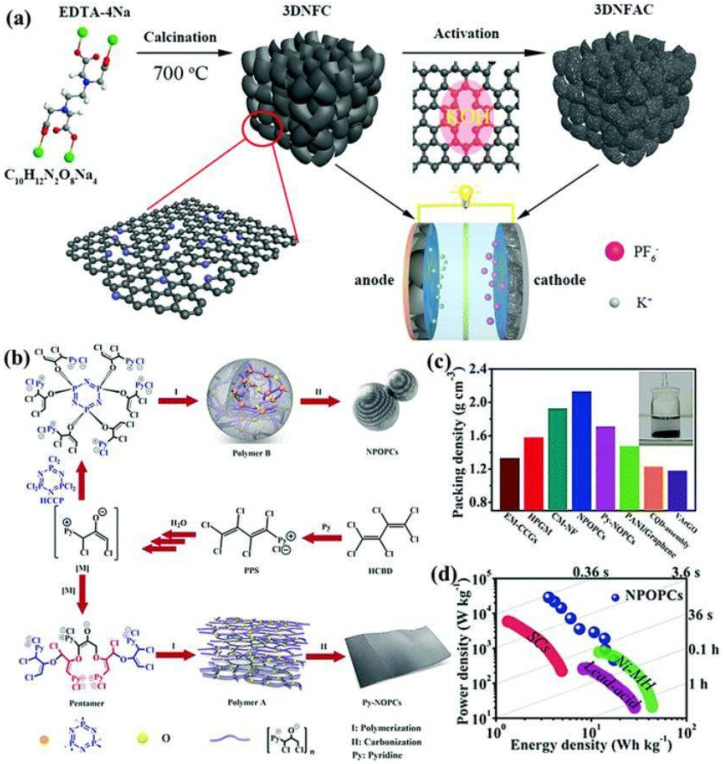
N-rich 3D hierarchical porous carbons shown schematically (**a**); the creation of porous carbons doped with several heteroatoms (**b**); packing rigor (**c**); Ragone plots (**d**). Reproduced with permission from [78].

Additionally, when tested as a supercapacitor, LHPC demonstrated excellent cycle stability (97% over 5000s) and a decent capacitance performance (165 F g ^−1^ at 0.05 A g ^−1^). Han et al. [75] and Jing et al. [76] fabricated N, O co-doped hierarchical porous carbon (HDPC) from pomelo peel as a biomass carbon source using ZnCl2 activation. The material exhibited a large specific capacitance of 180 F g^−1^ at 0.5 A g^−1^, with a capacitance retention of 75.6%, and showed large stability. Figure 9a,d show the coating of n-doped carbon on a self-produced Na_2_CO_3_ template [77].

Recently, 3D hierarchical porous carbon with large interlayer space and rich N content (5.74 at%) was fabricated by the direct calcination of tetrasodium ethylenediamine tetra acetic acid, wherein the decomposed C/N-containing skeleton of the salt precursor transformed into an N-doped carbon product coated on the self-generated Na_2_CO_3_ template.

## 5. Carbon Composite for Energy Storage

**Figure 10:**
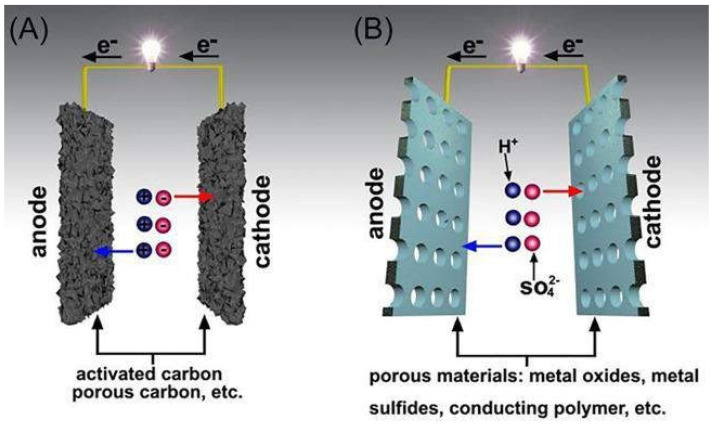
Schematic diagram of (**a**) an electrical double-layer capacitor (EDLC) and (**b**) a pseudocapacitor (PC). Reproduced with permission from [79].

**Figure 11:**
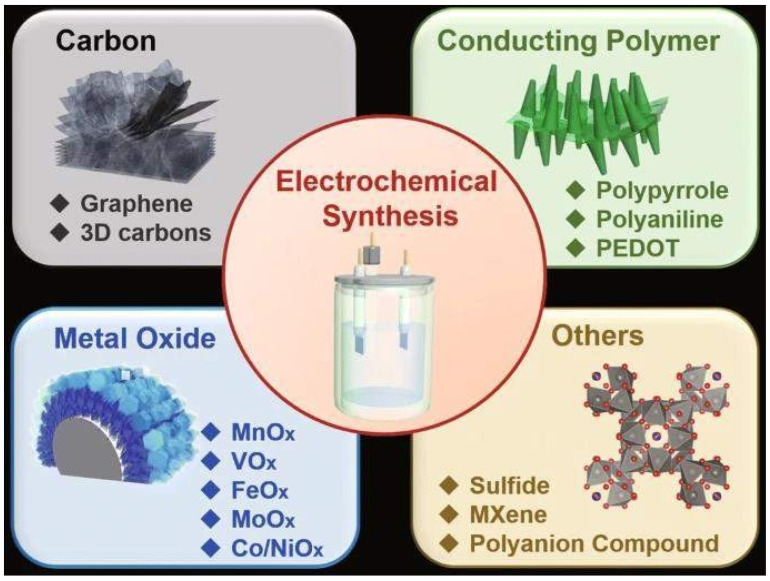
Progress of carbon-based materials. Reproduced with permission from [80].

Because of their unique structural, electrical, and mechanical qualities, carbon-based materials such as graphene, nitrogen-doped carbons, carbon nanotubes, and carbon quantum dots, can be employed as electrode material. However, as a result of their low volumetric capacitance, they are difficult to produce. Nanocarbons such as graphene or nanotubes can be incorporated to create mesoporous structures with a large specific surface area and remarkable resilience. This electrode appears to have significantly increased porosity for SC applications when different nanocarbons have been incorporated in activated carbon matrices. Figure 10 and Figure 11 show the progress of carbon-based material for energy-storage applications.

Carbon-based materials, for example, graphene, activated carbon, and carbon nanotubes have gained massive attention because of their essential electrical, thermal, and mechanical characteristics. Carbon materials as anode materials have some limitations because charge storage is bound through the adsorption–desorption of ions at the electrode/electrolyte interface, producing a double layer, and their collection, while synthesis and processing result in lower electrochemical activity.

### 5.1. Carbon–Metal Oxide Composites

The conductivity, cyclic capability, good mechanical strength, high capability, and current density of carbon and metal-based oxide materials are higher than those of other oxide materials [81]. This review also covers hitherto unexplored elements of carbon, MO, and transition-metal-based, supercapacitors, as well as synthesis techniques, properties, issues, and potential MO-C composites. In the comparison study, several supercapacitor attributes are examined, and the synthesis methods and performance of MO-C composites are fully detailed [82,83,84]. It is possible to conclude that MO-C composites have numerous features that make them good candidates for supercapacitor electrode materials.

Numerous metal oxides have been extensively explored in the areas of electrolytic supercapacitors and hybrid devices, including RuO_2_, TiO_2_, MnO_2_, Fe_3_O_4_, V_2_O_5_, NiO, and Co_3_O_4_.

Unlike in a capacitance charges storage device (EDL) using carbon materials, certain oxide nanoparticles, such as RuO_2_, MnO_2_, and Fe_3_O_4_, [84] display pseudocapacitance, in which very quick and reversible redox reactions take place on the electrode surface. In addition to activated carbon as an electrode material, other oxides [85] are used as elements in rechargeable battery electrodes that can be used in hybrid systems.

When metal oxides—so-called active materials—are introduced in sufficient quantities to the graphene structure, they can produce good electrode materials. Metal oxide nanoparticles operate as nanospacers between the graphene layers, preventing restacking. The flexible area between the 2D graphene sheets, on the other hand, provides a smooth horizontal path for the mobility of electrolyte ions, boosting the energy-storage ability.

**Figure 12:**
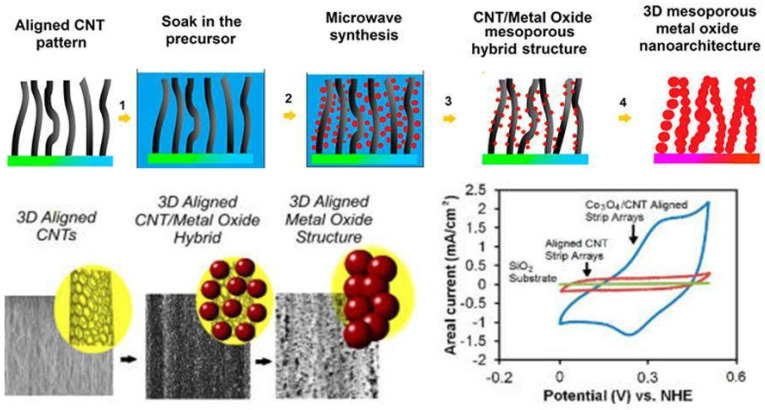
A schematic illustration of the experimental techniques used to create 3D mesoporous hybrid CNT/oxide architectures. Reproduced with permission from [86].

A synergistic impact is possible, and material costs can be decreased. The performance of supercapacitor electrodes is governed by the compositional constituents, microstructure, and physical properties of metal oxide–carbon composites. The cell performance is influenced by electrode porosity, electronic conductivity, pore-size dispersion, and specific surface area. Figure 12 shows a schematic illustration of the experimental techniques used to create 3D mesoporous hybrid CNT/oxide architectures.

**Figure 13:**
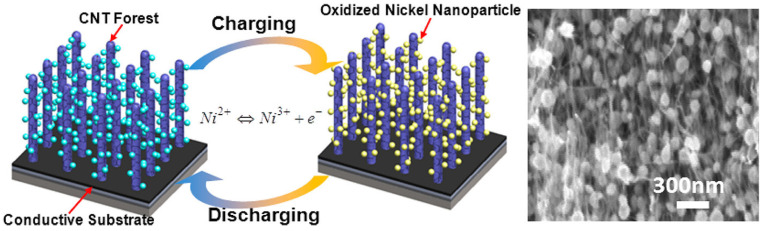
A schematic illustration of the experimental techniques used to create 3D mesoporous hybrid CNT/oxide architectures and 3D mesoporous metal oxide structures. Reproduced with permission from [88].

CNT is well-known for its exceptional electrical and mechanical capabilities, including a high length-to-diameter ratio and intrinsic metallic property [87]. It can also be used to create an ideal three-dimensional template known as a “CNT-forest” for depositing metal oxide to boost energy-storage capacity [88]. Figure 13 shows a schematic illustration of the experimental techniques used to create 3D mesoporous hybrid CNT/oxide architectures and 3D mesoporous metal oxide structures.

In pseudocapacitor applications, an Fe/Al/Mo stack layer that functioned as the catalyst and a conducting current collecting layer were used to grow nanotube (CNT) forests directly on a silicon substrate. The observed specific capacitance of 1.26 F/cm^3^ was found to be 5.7 times greater than pure CNT forest samples.

**Figure 14:**
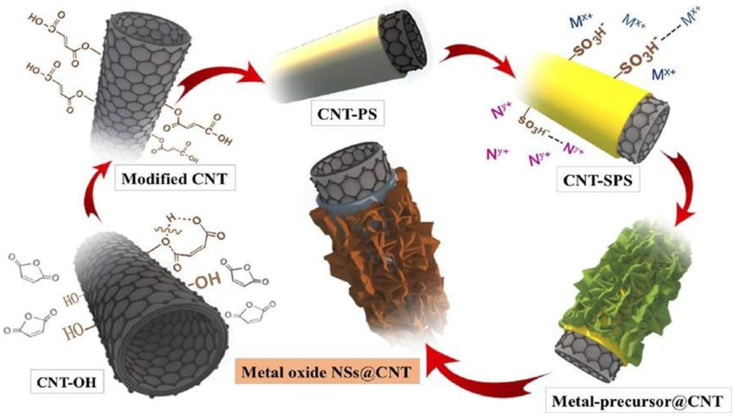
A synthetic approach for TMO@CNT hybrid materials, involving pre-coating CNT with sulfonated polystyrene, depicted schematically. Reproduced with permission from [89].

We present a straightforward synthetic method for coaxially growing TMO@CNT nanostructures with complete control over phase and morphology. When employed as electrode materials for lithium-ion batteries (LIBs) and electrochemical capacitors, these structures have a wide active surface and improved structural stability. Figure 14 represents this synthetic approach for TMO@CNT hybrid materials, which involves pre-coating CNT with sulfonated polystyrene, depicted schematically.

**Figure 15:**
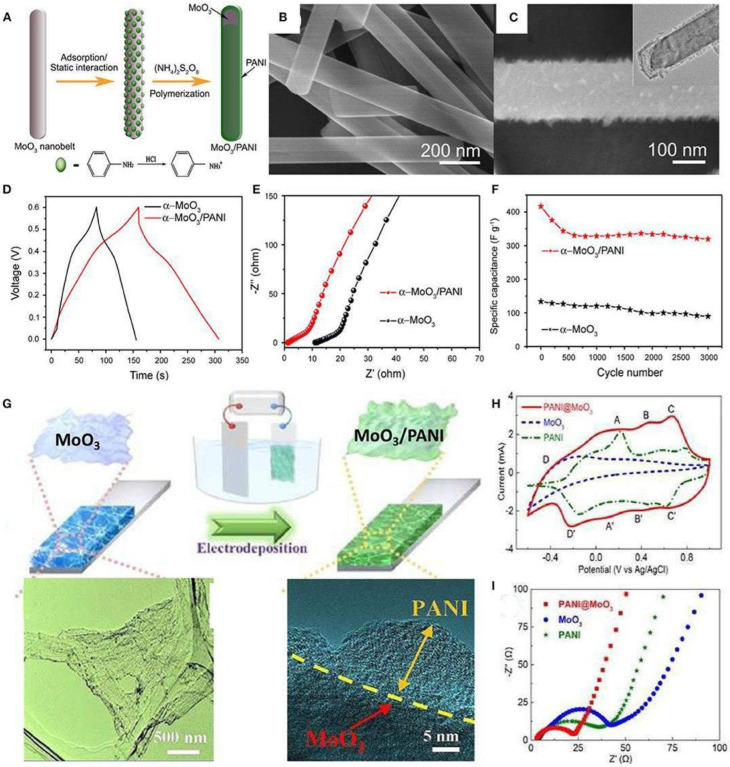
(**A**) Schematic illustration of the formation of the MoO_3_/PANI coaxial heterostructure nanobelts. (**B**) SEM images of the original α-MoO_3_ nanobelts. (**C**) SEM images and (inset) TEM images of the as-synthesized MoO_3_/PANI coaxial heterostructure nanobelts. (**D**) A comparison of the galvanostatic charge–discharge curves of the two comparative materials at a current density of 2 A/g. (**E**) EIS spectra comparison and (**F**) cycling performance at a scan rate of 50 mV/s of the two comparative materials. (**G**) Schematic illustration for the synthesis process of 3D MoO_3_/PANI hybrid nanosheet network film. (**H**) CV curves of MoO_3_, PANI, and 3D MoO_3_/PANI hybrid nanosheet network films in the potential range from −0.6 to 1 V at a scanning rate of 50 mV/s. (**I**) Nyquist plots of the MoO_3_, PANI, and 3D MoO_3_/PANI hybrid nanosheet network films. Reproduced with permission from [90].

Researchers have also worked on conjugated polymers and other materials. Figure 15 shows the creation of MoO3/PANI coaxial heterostructure nanobelts depicted schematically. This study [90] examines the development of energy-storage material, hierarchical structure topologies, and controlled electrical properties.

**Figure 16:**
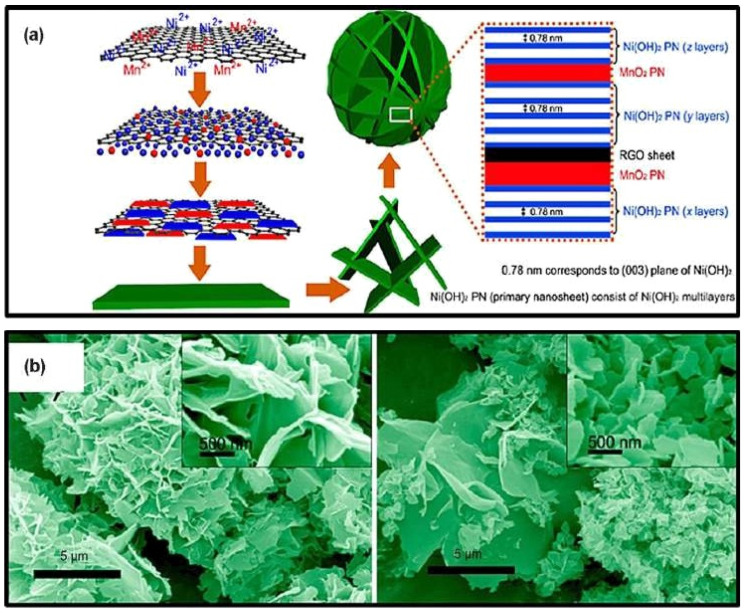
(**a**) A schematic depiction of a potential formation pathway for a Ni(OH)2-MnO2-rGO hybrid sphere, with (**b**) SEM results. Reproduced with permission from [91].

The heteroatoms have been doped on graphene to increase their intrinsic properties. Ma et al. explained the production of Fe2O3 supported by a hydrogel of nitrogen-doped graphene (Fe2O3/N-rGO), as illustrated in Figure 16 [91,92]. The SC was about 350 F g^−1^ at a current density of 10 A g^−1^. The capacity retention was measured to be 56.7% after 5000 cycles. Figure 16a shows the schematic depiction of a potential formation pathway for a Ni(OH)2-MnO2-rGO hybrid sphere, while Figure 16b shows the SEM results.

### 5.2. Carbon Metal Sulfide Composites

The study of innovative materials for energy-storage devices has gained popularity around the world. Because of their distinct and promising features, metal sulfides (MSs)-based materials have been proposed for ESs applications. Several research articles on MSs-based electrodes for ESs with improved performance have recently been published [93,94]. Carbon-based materials and metal sulfides have attracted considerable interest because of their exceptional characteristics and wide range of applications.

**Figure 17:**
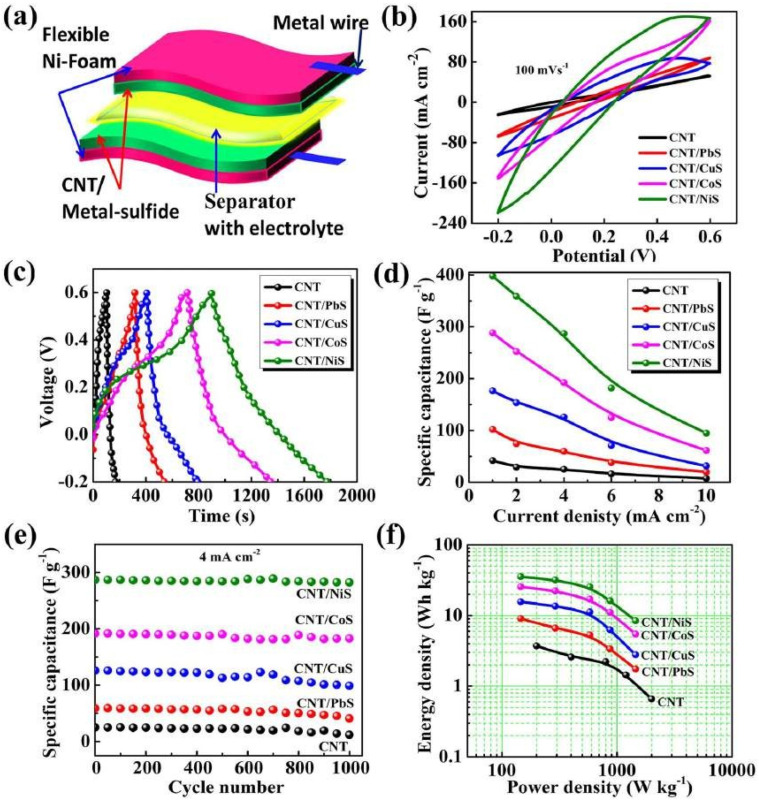
(**a**) Schematic of the flexible symmetric supercapacitor. (**b**) CV graphs for symmetric CNT/metal-sulfide supercapacitor devices at 100 mV s^−1^ in polysulfide electrolyte. (**c**) Charge-discharge curve at 1 mA cm^−2^, (**d**) specific capacitance versus current density, (**e**) stability performance and (**f**) Ragone plot for symmetric CNT/metal-sulfide supercapacitor cells. Reproduced with permission from [94].

Figure 17a depicts a flexible symmetric supercapacitor design. Figure 17b depicts typical CV scans of CNT and CNT/metal sulfide samples spanning a potential range of 0.2 to 0.6 V at a scan rate of 100 mV s^−1^. With an increasing scan rate, the CV areas of the electrodes rose, showing good capacitance retention [95,96]. The specific capacitance of the CNT/NiS device was 398.16 F g^−1^ at 1 mA cm^−2^ from the GCD curve (Figure 17d). These findings indicate that the specific surface area and mesoporous structures of this design contribute to a high supercapacitor performance.

**Figure 18:**
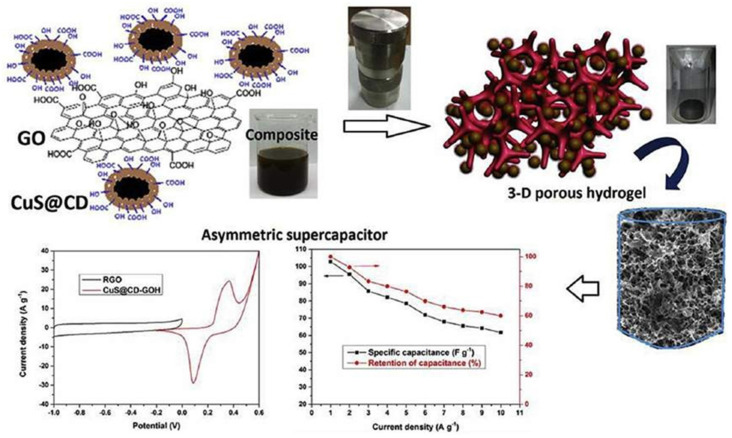
Carbon dot/copper sulfide nanoparticles-adorned GO hydrogel for storage applications. Reproduced with permission from [97].

Figure 18 represents a carbon dot/copper sulfide nanoparticles-adorned GO hydrogel for storage applications. A simple hydrothermal procedure at 180 °C was used to create CD-coated CuS (CuS@CD)-adorned GO hydrogels (CuS@CD-GOH), which were then optimized using various electrochemical investigations. CD worked as a stabilizer for the CuS nanoparticles, and at a current density of 1 A g^−1^ the CuS@CD-GOH had a high specific capacitance of 920 F g^−1^. In comparison to previously published studies of CuS and composite GO hydrogel-based supercapacitors, the findings are good. As a result, this research will shed new light on CuS and GO-based materials for storage applications.

**Figure 19:**
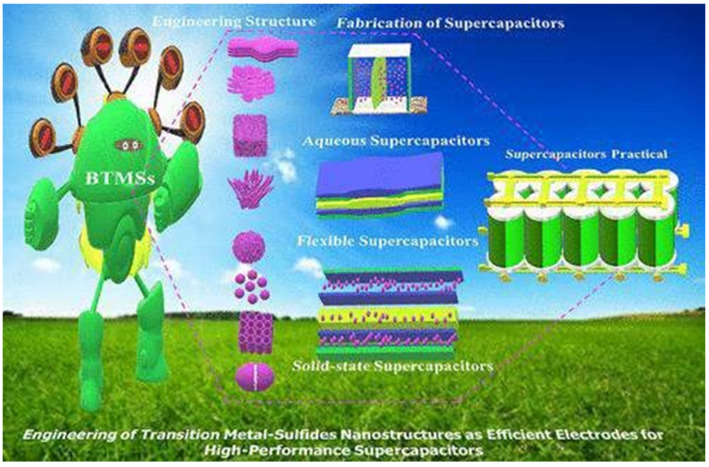
Efficient Electrodes for high-performance supercapacitors using transition-metal sulfide nanostructures. Reproduced with permission from [98].

Transition-metal-based electrode materials (TMEMs) are easy to synthesize in high yield from cheap and plentiful resources. In comparison to other TMEMs, binary transition-metal sulfides (BTMSs) have higher storage capacity, stronger electrical conductivity, more outstanding redox characteristics, and higher rate capability. Figure 19 is a representation of efficient electrodes for high-performance supercapacitors engineered using transition-metal sulfide nanostructures [95].

**Figure 20:**
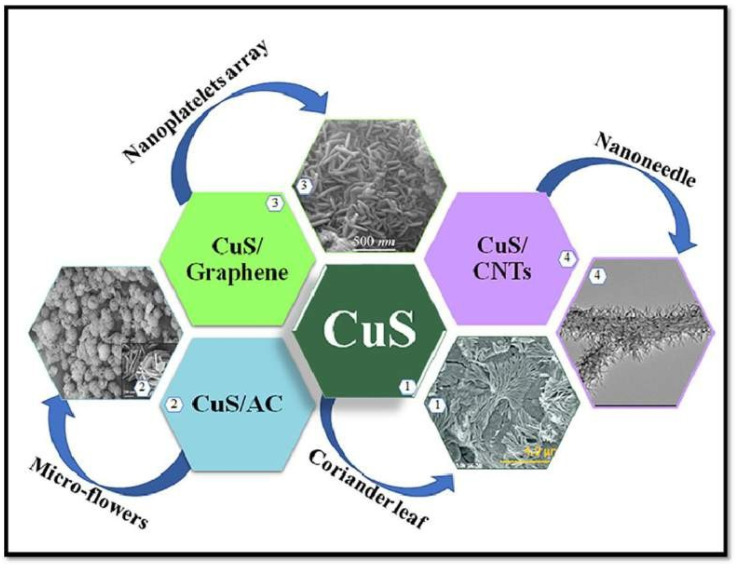
High-energy supercapacitor applications that are driving the development of carbon-based copper sulfide nanocomposites. Reproduced with permission from [94].

The current work provides in-depth assessments of recent developments in carbon materials, with an emphasis on copper sulfide nanocomposites made of carbon and their uses in high-energy supercapacitors. In order to address the soaring demand for effective electrochemical energy-storage devices, copper sulfides, and their use in the enhancement of supercapacitors, have generated a great deal of interest. Figure 20 represents the high-energy supercapacitor applications that are driving the development of carbon-based copper sulfide nanocomposites.

**Figure 21:**
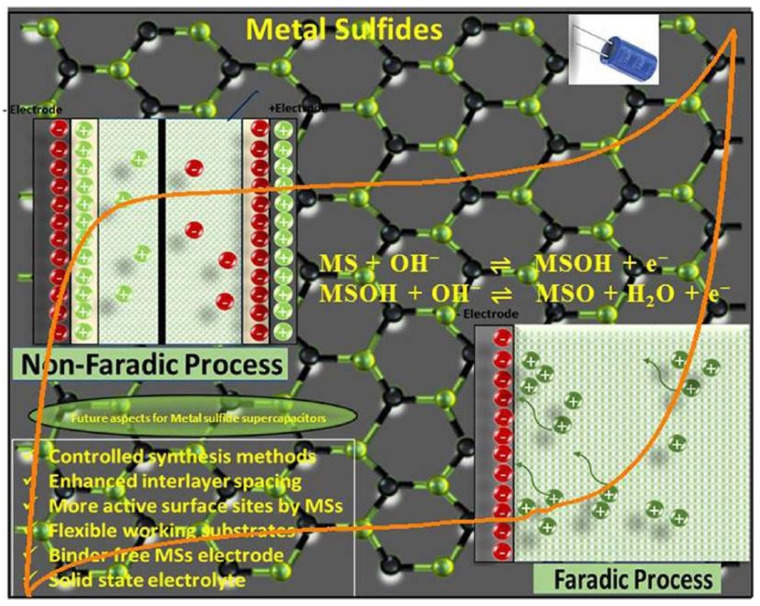
Challenges and opportunities for metal sulfide materials in supercapacitors. Reproduced with permission from [95].

Figure 21 shows the challenges and opportunities for metal sulfide materials in supercapacitors.

Drawing on a few research studies published in the literature [99], we propose the following ways to address the aforementioned concerns:

- To improve interlayer spacing, a composite of MSs with MXene and MOFs would be a wonderful concept [100,101];
- Mixed MOs created by the fizz mechanism or the deformed-metal-layered double hydroxides framework offer a more active surface area for the electrolyte and electrode as well as enough voids for quick ionic diffusion. This not only makes electrochemical reactions easier, giving greater electron mobility, but also makes rich redox reactions possible;
- The invention of carbon MSs composite materials may improve electric conductivity and buffer volume fluctuation and limit exfoliated MSs aggregation.

### 5.3. Carbon Conducting Polymer Composites

Redox-active conducting polymers (CPs) are attractive materials for application because they combine metal-like electrically conductive qualities with polymer-like mechanical capabilities. However, they function poorly because of volume changes throughout the charge and discharge cycles.

**Figure 22:**
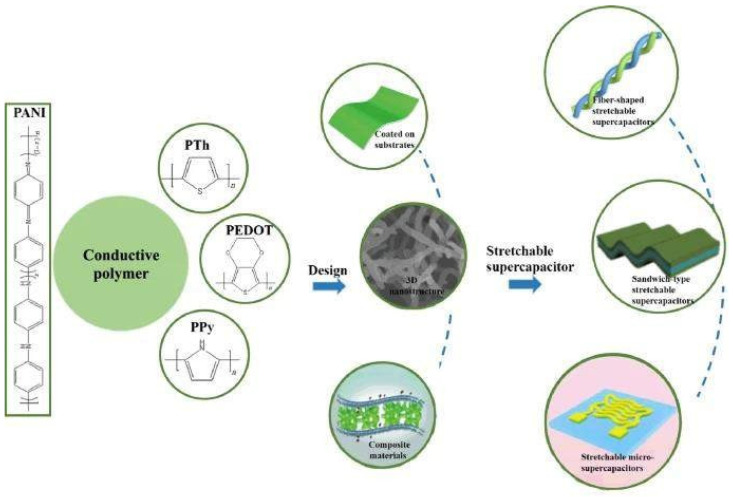
Stretchable supercapacitors utilizing conductive polymers. Reproduced with permission from [102].

Stretchable energy-storage systems that can operate continuously under high mechanical strain have become increasingly significant with the development of stretchable electronic gadgets. Stretchable supercapacitors (SSCs) are regarded as one of the most promising power sources for stretchable electronic devices due to their high power density, low energy density, and exceptional mechanical qualities. Figure 22 shows stretchable supercapacitors utilizing conductive polymers.

**Figure 23:**
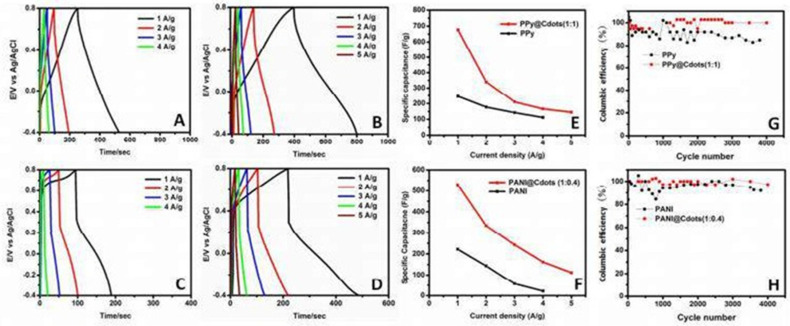
GCD curves of (**A**) PPy, (**B**) PPy@Cdots(1:1), (**C**) PANI, and (**D**) PANI@Cdots(1:0.4) at different current densities specific capacitance of (**E**) PPy and PPy@Cdots(1:1), and (**F**) PANI and PANI@Cdots(1:0.4). Coulombic efficiency of (**G**) PPy and PPy@Cdots(1:1) and (**H**) PANI and PANI@Cdots(1:0.4). Reproduced with permission from [103].

The galvanostatic charge and discharge (GCD) properties of PPy, PPy@Cdots, PANI, and PANI@Cdots were examined at varied current densities with potential ranges of -0.4 to 0.8 V to confirm the charge capacity of the as-prepared composites, as shown in Figure 23. At 1 A/g, the predicted capacitance was 245 and 222 F/g, respectively, for pristine polymers (PPy and PANI) and 676 and 529 F/g, respectively, for PPy@Cdots and PANI@Cdots(1:0.4).

**Figure 24:**
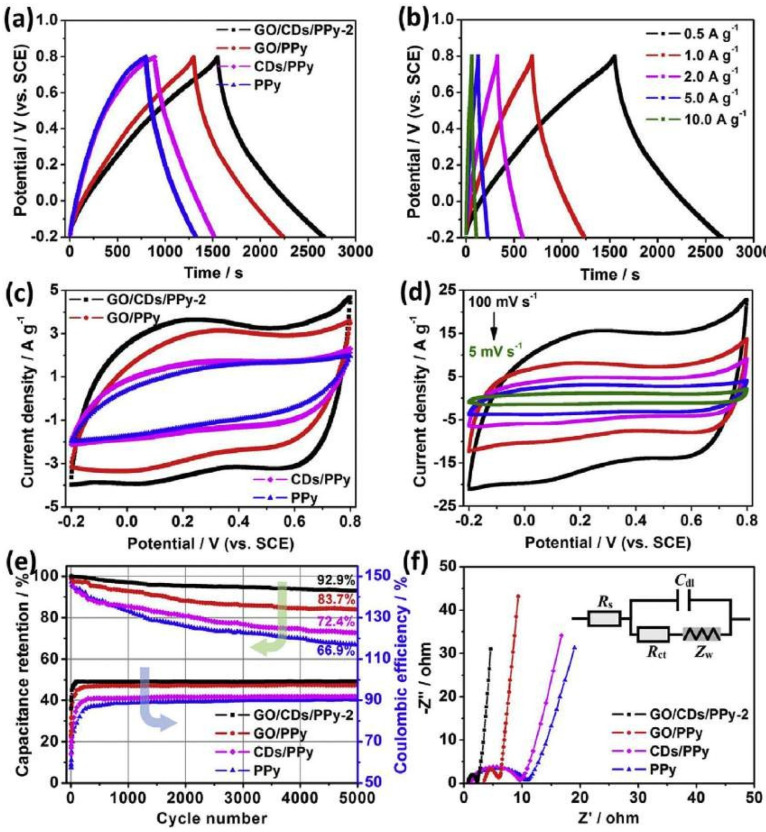
Investigation of the electrochemical properties of PPy, CDs/PPy, GO/PPy, and GO/CDs/PPy composites: (**a**) GCD curves at 0.5 A g^−1^ current density; (**b**) GO/CDs/PPy GCD curves at various current densities; (**c**) CV curves at 10 mV s^−1^ scan rate; (**d**) GO/CDs/PPy CV curves. Reproduced with permission from [104]; (**e**) Cycling stability; (**f**) Nyquist plots.

The GCD curves can be used to compute the specific capacitances of active materials on a single electrode (Cs). PPy, CDs/PPy, and GO/PPy have computed Cs values of 267, 305, and 468 F g^−1^, respectively. This validates the presence of pseudocapacitance in the GO/CDs/PPy hybrid electrode. However, unlike a conventional pseudocapacitor, there are no visible redox peaks to be seen. Figure 24a–d show good and steady capacitive behavior under the operating voltage of 1.0 V. To evaluate cycle stability, the composites were cycled 5000 times at a current density of 10.0 A g ^−1^ (see Figure 24e,f).

**Figure 25:**
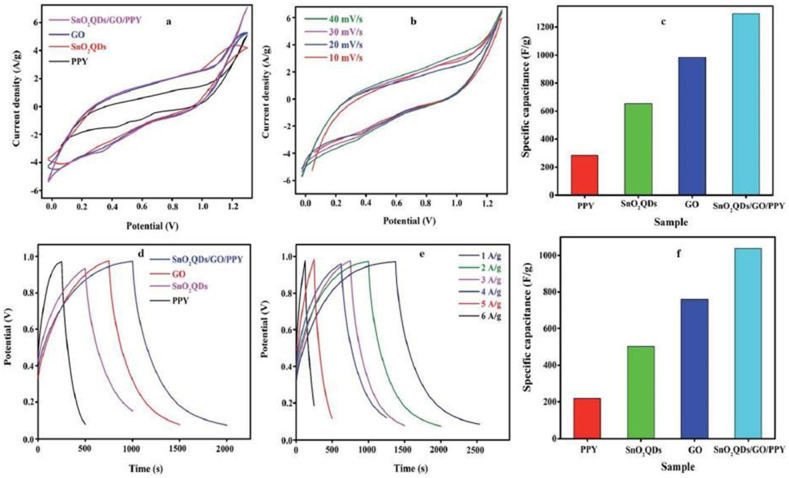
(**a**) CV curves of pure PPY, SnO2QDs, GO, and SnO2QDs/GO/PPY nanocomposites; (**b**) CV plot of SnO2QDs/GO/PPY nanocomposites; (**c**) cyclic voltammetry variation of Csp of various materials; (**d**) GCD of different nanocomposites; (**e**) GCD plots for SnO2QDs/GO/PPY ternary materials at different CD; (**f**) Csp of nanocomposites from GCD curve. Reproduced with permission from [105].

The inclusion of PPY and SnO2 in the compositegives both EDL capacitance and apparent pseudocapacitance, as demonstrated by the redox peak. The large-area redox peak is caused by the combined impact of SnO2, GO, and PPY. CV curves of the SnO2QDs/GO/PPY composite at scan speeds ranging from 10 to 40 mV s^−1^ are shown in Figure 25a–f. The maximum Csp estimated from the SnO2QDs/GO/PPY composite is 1296.14 F g ^−1^: as the scan rate increases, the Csp decreases, indicating a higher rate capacity.

### 5.4. Heteroatom-Doped Carbon Materials for Supercapacitor

**Figure 26:**
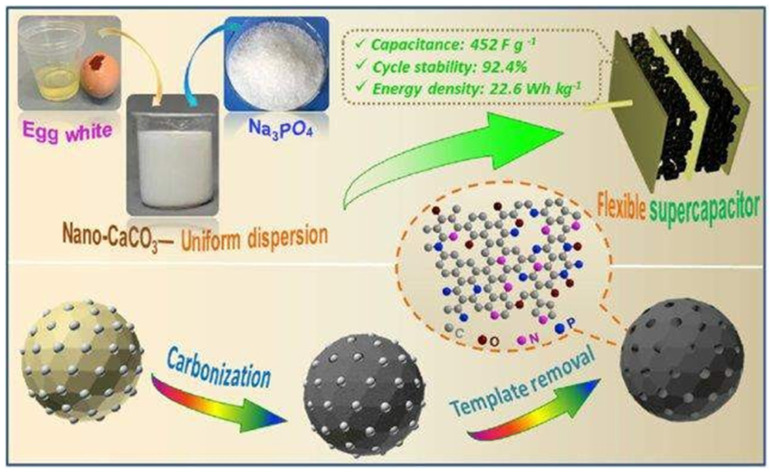
A schematic approach for synthesizing multi-heteroatom co-doped porous carbon for energy storage. Reproduced with permission from [107].

Heteroatom-doped carbon materials (HDCMs) have been the subject of extensive research as some of the most promising materials possibilities for usage in a variety of applications, including batteries, supercapacitors (SCs), and the oxygen reduction reaction (ORR) [106]. Figure 26 shows the schematic approach for synthesizing multi-heteroatom co-doped porous carbon for energy storage.

Porous carbon hybrids have recently received increased interest as electrode materials for supercapacitors; however, controlling their pore shape and element composition while maintaining optimal electrochemical performance remains a significant issue. As-prepared carbon has a high surface area (2576 m^2^ g^−1^), a well-balanced pore-size distribution with a substantial micropore volume (0.77 cm^3^ g^−1^), and a multi-heteroatom-doped carbon skeleton (3.9% N, 12.2% O, and 4.1% P).

**Figure 27:**
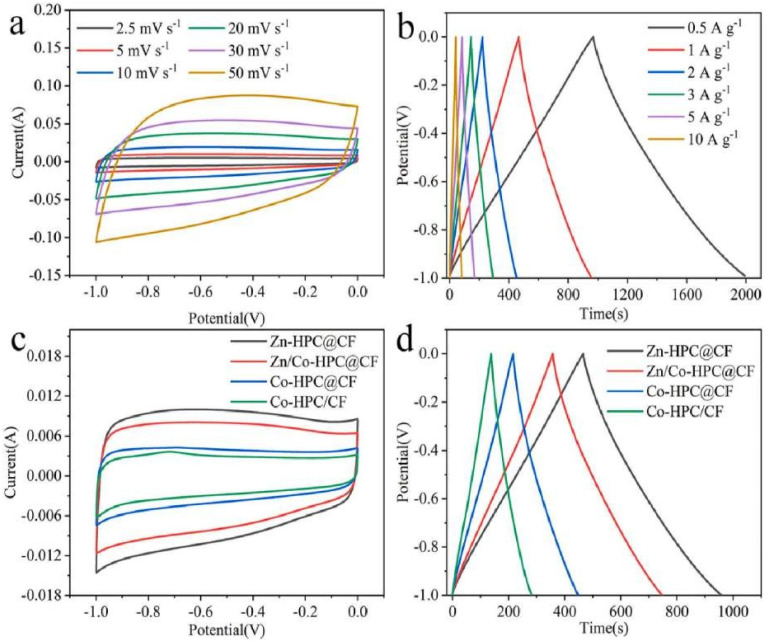
(**a**) CV curves at different scan rates and (**b**) GCD curves at different current densities of Zn-HPC@CF. (**c**) The CV curves at 2.5 mV s^−1^. (**d**) The GCD curves at 1 A g^−1^. Reproduced with permission from [108].

The CV curves for Zn-HPC@CF electrode material (Figure 27a,d) have an evident quasi-rectangular shape and strong symmetry at 50 mV s^−1^, which is typical EDLC behavior. The CV curves reveal no visible deformation at high scanning rates, confirming the material’s superior structural stability and reversible kinetics.

**Figure 28:**
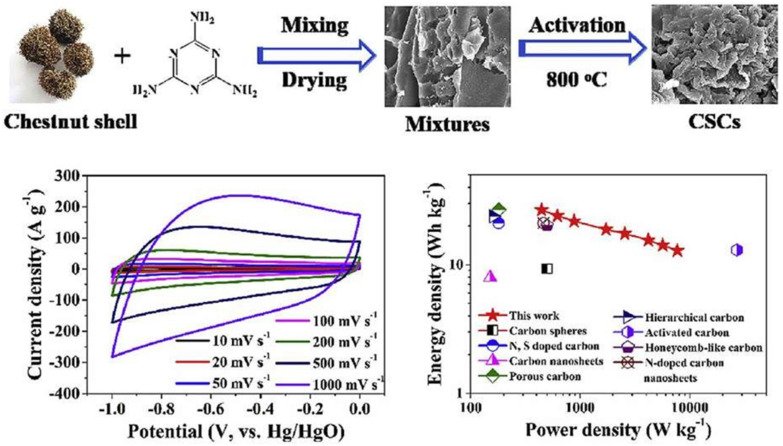
Superior performance in supercapacitors using multi-heteroatom-doped hierarchical porous carbon produced from chestnut shells. Reproduced with permission from [109].

Porous carbon materials made from biomass offer tremendous potential for usage in supercapacitors due to their inherent availability, low price, and high heteroatom concentration. A one-step carbonization method was used to produce multi-heteroatom-doped porous structure carbon using chestnut shells as the raw resources and melamine as the activator. Because the carbons generated from chestnut shells have a high specific area (691.8 m^2^ g^−1^) as shown in Figure 28, as well as many interconnected micropores/mesopores and a proper amount of graphitization, the constructed symmetric supercapacitor is an excellent power source.

**Figure 29:**
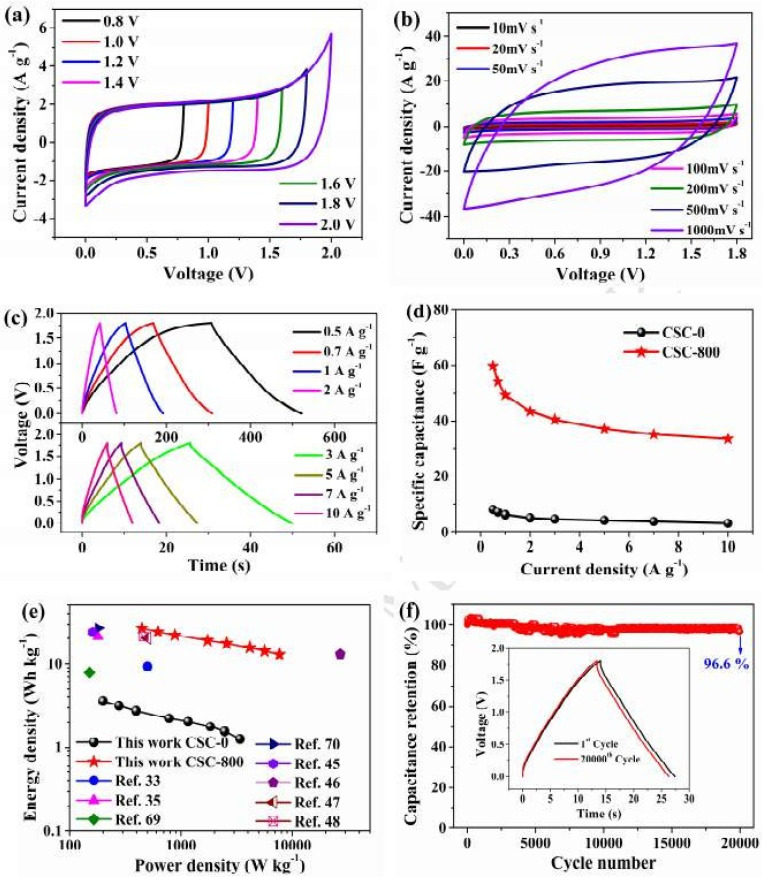
EC study of the CSC-800/CSC-800: (**a**) CV curves with different voltages; (**b**) CV curve with a range of scan rates from 10 to 1000 mV s^−1^; (**c**) GCD curves with a different CD; (**d**) Csp vs. CD; and (**e**,**f**) Ragone plots. Reproduced with permission from [110].

The CV curves of the CSC-800/CSC-800 symmetric supercapacitor keep a rectangular-like shape (Figure 29b) and show good rate capabilities [110,111]. The cell’s specific capacitance is 59.8 F g^−1^ at 1 A g^−1^ and remains as high as 33.6 F g^−1^ at 10 A g^−1^ (Figure 29d), which is significantly higher than that of previously reported biomass-derived carbon-based symmetric SCs (Figure 29a–f) [112].

## 6. Recent Advances and Trends

The rapid progress of modern electronics has left room for further breakthroughs in the development of high-energy, high-power, and highly stable energy-storage systems. Carbon-based supercapacitors, which can store electrical energy, are effective in this regard. However, due to restricted charge accumulation and slow mass dispersion, commercial SCs based on activated carbons have low energy densities in organic electrolytes. To address these challenges, significant efforts have been made to enhance the energy-storage capacity of SCs by exploring highly capacitive electrodes and electrolytes. This review outlines the challenges encountered during the development of electrode synergy and presents potential future directions for the next generation of SCs.

### 6.1. High Surface Area

The International Union of Pure and Applied Chemistry (IUPAC) classifies pores into three kinds based on their diameters: micropores (2 nm), mesopores (2–50 nm), and macropores (>50 nm) [113,114]. Micropores play an important role in providing a large accumulation platform for high-energy storage through molecular sieving and regulated diffusion effects.

**Figure 30:**
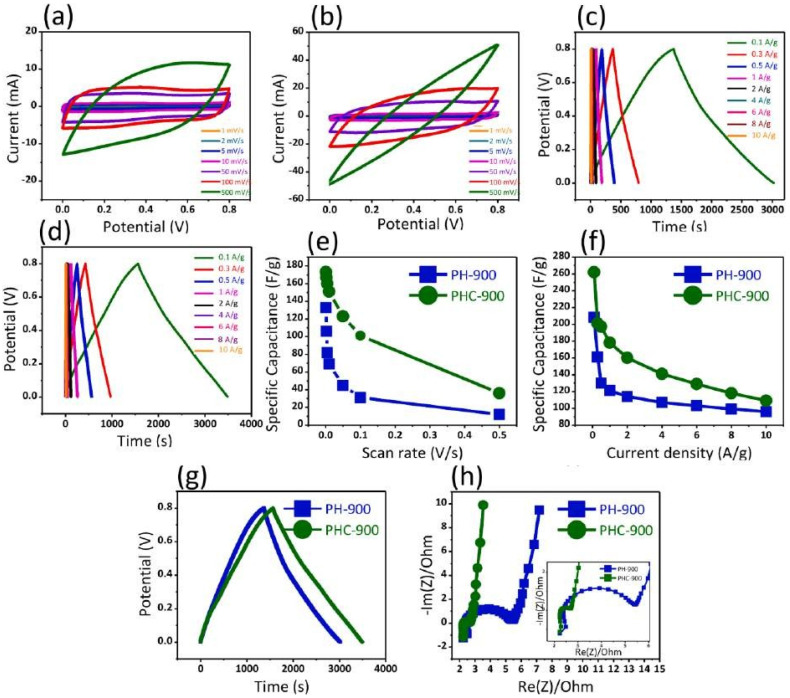
CV results of (**a**) PH-900 (**b**) PHC-900, GCD results of (**c**) PH-900 (**d**) PHC-900 (**e**) scan rate dependent specific capacitance and (**f**) current density dependant specific capacitance (**g**) comparison of GCD curves at 0.1 A/g current density (**h**) EIS spectra (inset at high resolution at the higher frequency range). Reproduced with permission from [114].

The CV curves of PH-900 and PHC-900 are shown in Figure 30a,b, respectively. The GCD plots of PH-900 and PHC-900 are shown in Figure 30c,d, respectively [115]. Both materials attained high specific capacitance at modest CD (0.1 A/g) (Figure 30f–h). All the carbon-based material has a very good surface area, exceptionally useful for storage application.

Hansa Mahajan et al. [116] developed a high-performance supercapacitor made of biocarbon-based MoS2 (Bio-C/MoS2) nanoparticles manufactured from date fruits using a simple hydrothermal method. We present here the high specific capacitance of a carbon-based nanocomposite created by pyrolysis, a technology for transforming agricultural biowaste into a highly economical energy supply.

### 6.2. Morphology Control

Recent research efforts have focused on leveraging the exquisite shape-dependent and defect-induced properties of electrode materials for spectacular device-performance gains. Among the different options for nano/micro morphology control, solution-based chemical processes provide paths for the creation of varied morphologies, enabling the usage of materials.

**Figure 31:**
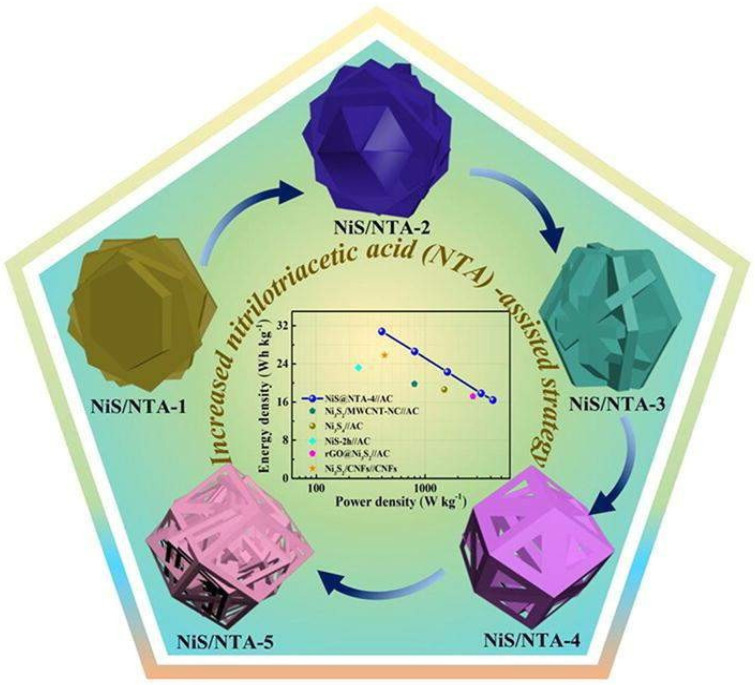
NiS/carbon hexahedrons produced from a nitrilotriacetic acid assembly for supercapacitors. Reproduced with permission from [117].

The improved NiS/carbon electrode (NiS/NTA-2) performs admirably in terms of capacitive performance, with an impressive capacity of maximum energy density and power density up to 35.1 Wh kg^−1^ and 4509.3 W kg^−1^, respectively, and equally impressive long-term stability of 87.2%. Figure 31 shows the NiS/carbon hexahedrons produced from a nitrilotriacetic acid assembly for supercapacitors.

**Figure 32:**
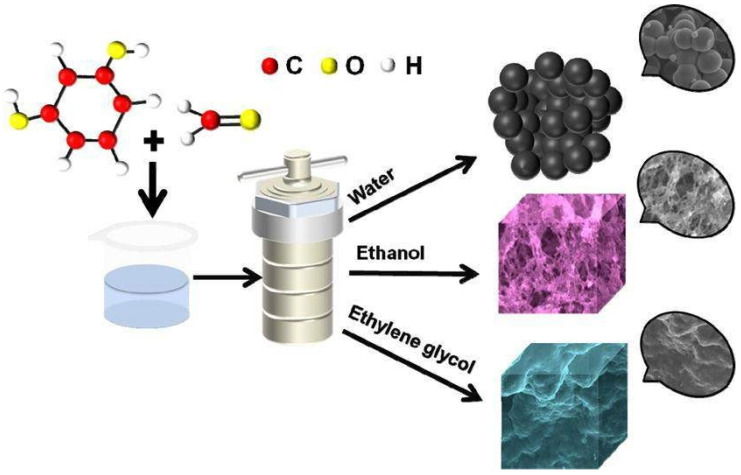
Controlled porous carbon synthesis and electrochemical performance of supercapacitors. Reproduced with permission from [118].

Porous carbon achieved a specific capacitance of 174 F g^−1^ at a current density of 1 A g^−1^ when utilized as the electrode material for a supercapacitor. The energy density of the supercapacitor device was 4.9 Wh kg^−1^ at a power density of 225.0 W kg^−1^ and 3.9 Wh kg^−1^ at a power density of 4457.1 W kg^−1^. After 10,000 cycles, the capacitance retention rate was 92.1%. Figure 32 shows the controlled porous carbon synthesis and electrochemical performance of supercapacitors.

### 6.3. Pore-Structure Regulation

The thermal stability of chili-straw biochar increases as the pyrolysis temperature rises. The testing of the electrochemical characteristics of the porous carbon revealed that PC500 has a high specific capacitance of 352 F/g at 1 A/g, whereas conventional heating has a specific capacitance of 226.1 F/g.

**Figure 33:**
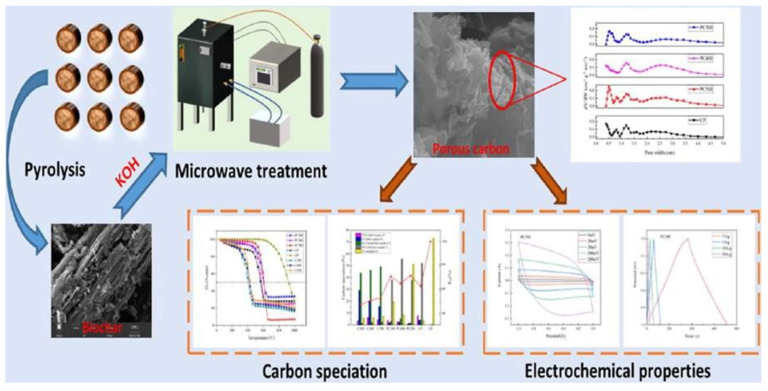
Microwave treatment of chili-straw pyrolysis residue yields high-value porous carbon. Reproduced with permission from [119].

Microwave porous carbon has superior electrical characteristics when compared with traditional porous carbon, and biochar generated at higher temperatures has a pore structure. Figure 33 shows how the microwave treatment of chili-straw pyrolysis residue yields high-value porous carbon.

**Figure 34:**
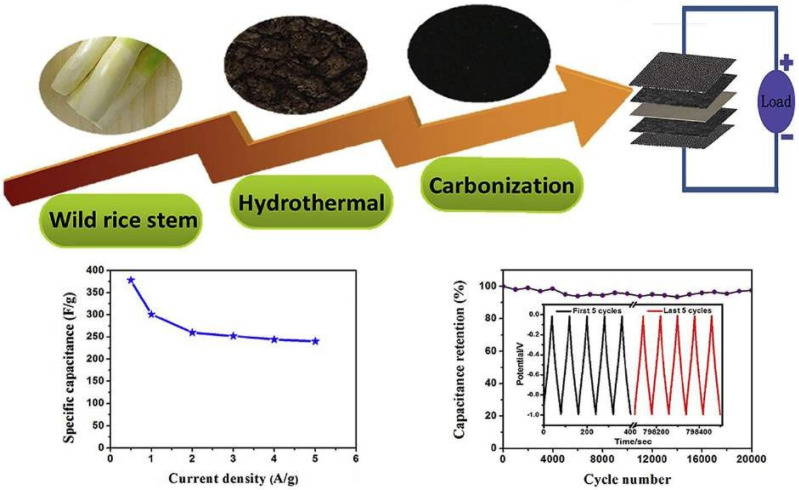
Porous carbon material originated from wild rice stem and used in supercapacitors. Reproduced with permission from [120].

In a three-electrode arrangement, activated porous carbon was synthesized at 800 °C with a 6 M KOH electrolyte. Furthermore, the constructed symmetric supercapacitor achieved a high energy density of 13.05 Wh/kg at a power density of 250 W/kg, showing good capacitive performance. Figure 34 shows the porous carbon material originated from wild rice stem and used in supercapacitors.

## 7. Conclusions

In response to the growing demand for energy-storage solutions, supercapacitors have become a prominent energy-storage device due to their high power density and long cycle life. Carbon materials are extensively used as electrode materials for supercapacitors, thanks to their large specific surface area, plentiful pore structure, excellent electrical conductivity, and chemical stability. To improve the electrochemical properties of carbon materials for supercapacitors, significant research has been conducted. As a common electrode material, carbon materials play a crucial role in supercapacitors. This review comprehensively covers the research progress of carbon-based supercapacitors, focusing on three main aspects:

(1) Recent advancements in the working mechanisms of energy-storage devices based on carbon-based materials.
(2) A range of frequently used carbon electrode materials for supercapacitors, describing their history, usage in supercapacitors, and advantages.
(3) The potential for future research and development in the field of carbon materials in supercapacitors, with the development and optimization of novel carbon nanomaterials offering new possibilities for hybrid supercapacitors.

While challenges remain in maximizing the carbon/electrolyte synergy, we are confident that high-energy, large-power, and long-lasting carbon materials will soon be realized for various commercial applications in electronics and other fields.

## Data Availability

Not applicable.
